# The Quest for the *Sli* Locus

**DOI:** 10.1007/s11540-024-09792-3

**Published:** 2024-09-13

**Authors:** Ernst-Jan Eggers, Ying Su, Sjaak A. W. van Heusden, Michiel E. de Vries, Christian W. B. Bachem, Richard G. F. Visser, Pim Lindhout

**Affiliations:** 1 Solynta, Dreijenlaan 2, 6703 HA Wageningen, The Netherlands; 2 Graduate School Experimental Plant Sciences, Wageningen University & Research, Wageningen, The Netherlands; 3 Plant Breeding, Wageningen University & Research, PO Box 386, 6700 AJ Wageningen, The Netherlands; 4 Present Address: McCain Global Agriculture, Florenceville, New Brunswick Canada

**Keywords:** Development of mapping populations, Diploid potato breeding, Genetic analysis, Phenotyping protocol, Self-compatibility

## Abstract

Genetic gain in potato breeding is limited by the heterozygous tetraploid genome of cultivated potato. Recent efforts to breed potato at the diploid level promise to improve genetic gain and allow more straightforward genetics and introgression breeding. Diploid F1 hybrid potato breeding relies on the ability to create diploid inbred lines via repeated self-fertilization. However, self-fertilization of diploid potato is hampered by a gametophytic self-incompatibility system encoded by the S-locus that prevents fertilization by self-pollen. Nonetheless, self-compatible diploid potato genotypes exist and have been used to create inbred lines. The *S-locus inhibitor* (*Sli*) gene is a dominant gene that provides strong self-compatibility in diploid potato and was previously mapped to Chromosome 12. While the *Sli* gene has already been identified and characterized, the most tedious challenge was to develop the optimal phenotyping methods and genetic populations preceding the cloning of this gene. To this end, we developed an effective phenotyping protocol to identify suitable parents and create diploid populations segregating for *Sli*. We show that an accurate phenotyping method is crucial to discriminate between confounding fertility factors and self-compatibility. In addition, we found that the *Sli* locus shows extreme segregation distortion on Chromosome 12. Finally, we used these insights to develop three F1 populations that segregate for *Sli*, which we later used for the identification of the *Sli* gene.

**Supplementary Information:**

The online version contains supplementary material available at 10.1007/s11540-024-09792-3.

## Introduction

Potato (*Solanum tuberosum*) is the third most important food crop after rice and wheat. Although the yield in these cereals has increased annually with 1–2%, genetic gain in potato has been limited or absent over the last century. This is mainly due to characteristics of the current potato breeding system. A typical breeding programme starts with an initial cross between two tetraploid parents. The F1 progeny populations are vegetatively multiplied as clones and phenotypically assessed over multiple years, resulting in a limited number of well-performing genotypes. However, due to the high allelic diversity, including deleterious alleles that are hidden in the multiple genomes in tetraploid potato, the F1 populations show tremendous segregation of traits and large populations are required to identify clones that combine the desired traits of the parents, and show a yield performance that is at least similar to if not better than the parental genotypes (Lindhout et al. 2011).

Recently, an alternative breeding strategy has been developed based on the self-fertilization of self-compatible (SC) diploid genotypes to create homozygous inbred lines, which can then be crossed to produce homogenous F1 hybrids (Lindhout et al. 2011; Jansky et al. 2016). Near-homozygous diploid inbred lines are the result of multiple rounds of self-fertilization, each of which offers the opportunity to expose homozygous deleterious alleles, while meiotic recombination may break undesired negative linkages and lead to desired combinations of linked traits enabling the selection of favourable genotypes. This has sparked interest in the mechanisms of sexual reproduction in potato (Plantenga et al. 2019; Seibert et al. 2020; Bethke and Jansky 2021).

Most diploid potato lines are naturally self-incompatible (SI) due to the Gametophytic Self-Incompatibility (GSI) system, encoded by the *S-locus*. The potato *S-locus* encodes S-RNases that are expressed in stylar tissues, which are toxic to pollen tubes unless the pollen expresses S-locus F-Box (SLF) proteins that can recognize and detoxify the S-RNases. Each S-allele encodes one S-RNase and multiple SLFs that together can recognize all S-RNases except the one encoded on the same S-allele (Kubo et al. 2010).

While most diploid potatoes are self-incompatible, most tetraploid potatoes are self-compatible due to the heteroallelic pollen effect (McClure et al. 2011). Tetraploid genotypes that have at least two different S-alleles produce diploid pollen, of which at least 25% express two different S-alleles and are thus able to detoxify all S-RNases, resulting in self-compatibility. When diploids are generated from such tetraploids via anther culture or prickle pollination, they lose the heteroallelic pollen effect and become self-incompatible because diploids produce haploid pollen that express only one S-allele. However, rare examples of self-compatible diploid potatoes have been described by several authors. Olsder and Hermsen identified two self-compatible dihaploids, G254 and B16, derived from tetraploid cultivars Gineke and Black 4495, respectively (Olsder and Hermsen 1976). They concluded that these genotypes harbour a genetic system that overcomes self-incompatibility. Dihaploid G254 has since then been extensively used in diploid breeding programmes at Wageningen University & Research, and one of the many descendants of G254, RH89-039–16, has been used as reference genotype around the world for genetic studies and genome sequencing (Xu et al. 2011; Peterson et al. 2016; Zhang et al. 2019; Clot et al. 2020; Zhou et al. 2020). Similarly, Peloquin and Hougas obtained a self-compatible dihaploid, US-W4, from the tetraploid clone Minn. 20–20-34 (Peloquin and Hougas 1960). This diploid genotype, US-W4, has also been widely used in breeding and research programmes (De Jong and Rowe 1971; Jansky 2011; Braun et al. 2017; Marand et al. 2017; Kaiser et al. 2021; Bamberg et al. 2021; Song and Endelman 2023).

Later, Hosaka and Hanneman identified a self-compatible accession of *S. chacoense* (chc 525–3) and mapped the “*S*-locus inhibitor (*Sli*) gene” on Chromosome 12 in an F1 population derived from a complex interspecific pedigree (Hosaka and Hanneman 1998a, b). They mapped the *Sli* locus at the telomeric end of the long arm of Chromosome 12, at 10-cm distance from markers CT156E5120 and 89–1320. Interestingly, they observed segregation distortion in this region and suggested that pollen carrying *Sli* may have a gametophytic advantage during the fertilization process. However, based on the observation of phenotypic segregation for self-berry set in the S8 generation, Hosaka and Hanneman suggested that *Sli* acts *sporophytically*, meaning that the genotype of the *sporophyte* rather than the haploid genotype of the *gametophyte* determines the outcome of the pollination, and that homozygosity for *Sli* itself is lethal or that it is closely linked to a lethal allele.

The location of *Sli* was later confirmed by Peterson et al. (2016) who mapped self-compatibility in an F1 population derived from the cross DM × RH. Remarkably, they observed highly significant associations between self-compatibility and 88 SNPs on the long arm of Chromosome 12, but this association was absent in the S3 generation, suggesting that this locus was fixed in the S3^7^. Further confirmation of the location of *Sli* was provided by Clot et al., who used Comparative Subsequence Sets Analysis (CoSSA) to narrow down the location of *Sli* to a 333 kB interval on the distal end of the long arm of Chromosome 12. Furthermore, Clot et al. showed that the *Sli* specific haplotype is present in a wide variety of tetraploid potato cultivars, a feature that they attribute to the presence of the clone *Rough Purple Chili* in pedigrees of many American and European varieties (Clot et al. 2020).

Using the *Sli* gene, Hosaka et al. generated nearly homozygous potato inbred lines (Phumichai et al. 2005; Hosaka and Sanetomo 2020). Similarly, Jansky et al. generated the M6 line that has since then been used as a reference genotype and parent for diploid mapping populations (Jansky et al. 2014; Endelman and Jansky 2016; Leisner et al. 2018; Marand et al. 2019). Recently, Solynta and Wageningen University & Research released the genome sequence of the inbred line Solyntus and Solynta released this genotype to the academic community where it is now being used in labs around the world for potato genetic research (van Lieshout et al. 2020; Freire et al. 2021; Hosaka et al. 2022).

Most results so far point into the direction of the *Sli* locus at the distal end of Chromosome 12. However, the exact position and the identity of the *Sli* gene still remain obscure. Here we report on the quest for *Sli* in a detailed genetic study. As a first step, we define the most accurate protocol to phenotype the self-compatibility that is associated with the *Sli* allele. Next, we select the most suitable parents for genetic studies. Lastly, we show that *Sli* inherits gametophytically, and we develop populations segregating for *Sli*. In this way we show the gametophytic inheritance of the trait which opened the route to the molecular cloning and characterization of the responsible gene (*Sli*) from the distal end of Chromosome 12.

## Materials and Methods

### Plant Materials

**Fig. 1:**
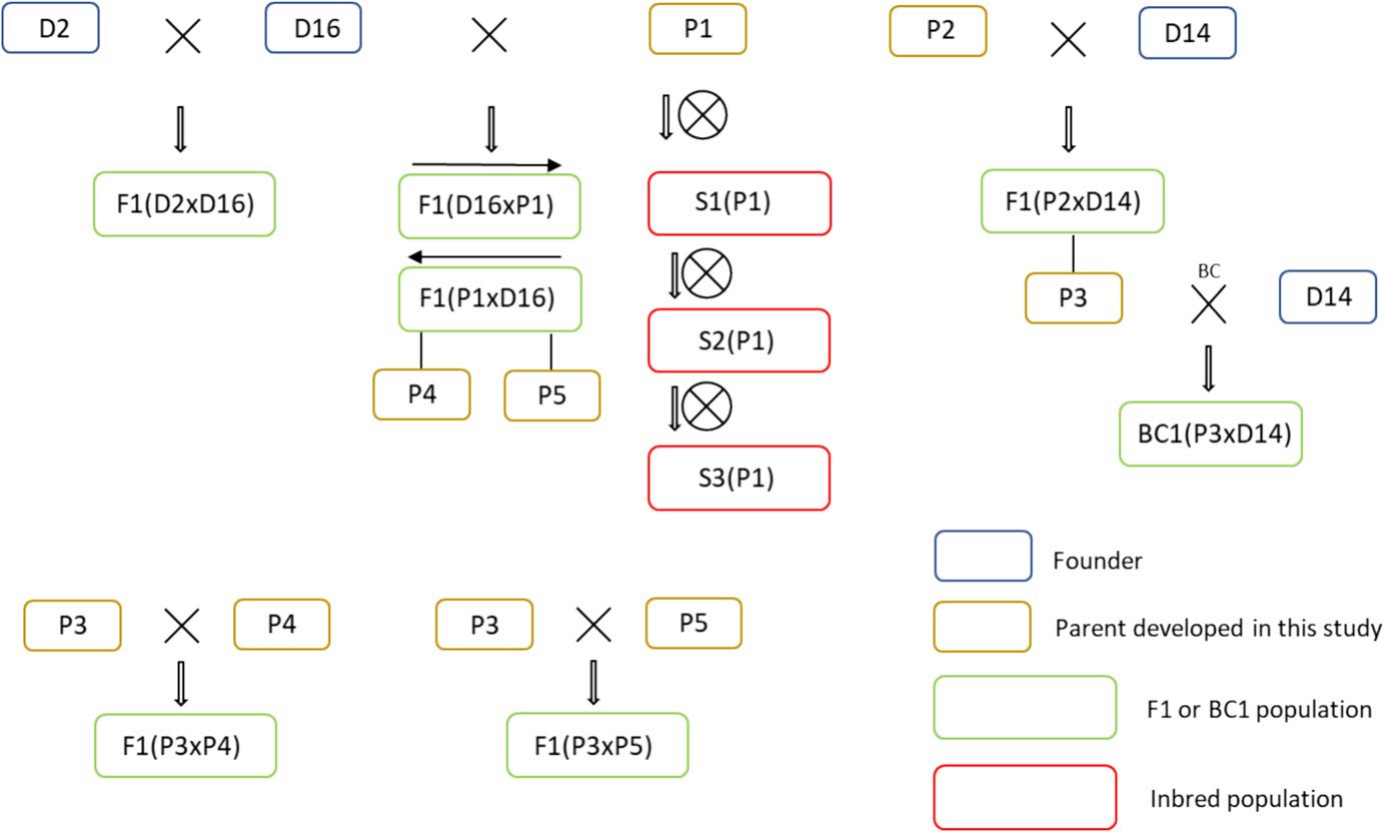
Pedigree relationships of the materials used in this study

In this study, we used a total of 19 populations of diploid potato material from *S. tuberosum* background derived from five parental clones from the Solynta breeding germplasm (Lindhout et al. 2011; Meijer et al. 2018). All parental genotypes and populations are listed in Supplementary Tables 1 and 2, and the pedigree information is shown in Fig. 1.

### Greenhouse Conditions

Potato seeds were sown in seed trays in the greenhouse, and 5-week-old seedlings were transplanted into pots. All plants were grown in greenhouses that were heated when the temperature dropped below 14 °C and cooled by opening the windows when the temperature rose above 19 °C. Artificial lighting was used to supplement the natural light when the light intensity outside dropped below 85 W/m^2^. Plants were grown in a special potato substrate mix from Lentse Potgrond (Lentse Potgrond B.V, Katwijk, the Netherlands). The used substrate mix is composed of a peat-mixture for balanced water uptake, basic slow-release fertilizer and lime to ensure the required pH level. The substrate mix was fertilized using a 20:20:20 nitrogen:phosphorus:potassium solution with an electrical conductivity (EC) of 1.5 which was supplied to the plants using a drip system. The amount of the solution given via the drip irrigation was adjusted to the growing stage of the plants, with small plants receiving about 60 ml per day and large plants receiving up to 720 ml per day. Depending on the greenhouse climate conditions, additional manual watering was applied when required.

### Evaluation of Self-Compatibility

Male fertility was assessed by collecting pollen from multiple flowers per plant in a micro-centrifuge tube and scoring the amount of released pollen on a scale from 0 to 3 (where a score of 0 means no pollen was released, and a score of 3 means abundant pollen was released). Female fertility was assessed by bulking pollen from at least three unrelated plants and using this bulk pollen to pollinate the plants under investigation. Successful fertilization requires that pollen tubes grow through the stylar tissue until the ovules. Therefore, we analysed pollen tube growth in pollinated pistils using fluorescence microscopy.

Styles from self- and cross-pollinated flowers were detached from the ovaries 48 h after pollination, and pollen tube growth was imaged using a fluorescence microscope. Plants that set more than one self-berry containing at least 35 seeds per berry were considered self-compatible. This lower limit is based on rare observations of seeds in berries in self-incompatible controls, which were set without manual pollination. Plants were declared self-incompatible when:

1. No self-berries were set after ten manual self-pollinations.
2. Berries were set after crossing with bulked pollen.
3. Pollen tube growth arrested before the ovaries had been reached in self-pollinated styles as observed by using fluorescence microscopy (Eggers et al. 2021).

### DNA Extraction, SNP Selection and KASP Analysis

Leaf discs from population S1(P1) were sampled into 96 well plates. DNA was extracted using sbeadex™ (LGC Genomics GmbH, Berlin, Germany) by VHLGenetics (Wageningen, the Netherlands) according to the manufacturer’s protocol. To design KASP markers, P1 was sequenced with Illumina 150 nt PE with approximately 20 × coverage, reads were mapped to DM4.03 and variant calling was performed by ServiceXS (Leiden, the Netherlands). For selected SNPs on Chromosomes 1 and 12, Kompetitive allele-specific PCR (KASP™) was performed by VHLGenetics (Wageningen, the Netherlands) according to the protocol provided by the manufacturer (LGC Genomics GmbH, Berlin, Germany). The quality of the resulting KASP marker data was assessed using SNPviewer (available at lgcgroup.com/products/genotyping-software/snpviewer); markers that did not segregate or showed unexpected segregation were discarded from the analysis. An overview of the markers and their genotypes can be found in Supplementary Table 3.

### Linkage Analysis and QTL Mapping

Each genotypic call was assigned to either haplotype 1 or 2 of parent P1 using the recombination rates between the markers. All the marker data was converted to ahb coding (where *a* means homozygous haplotype 1, *h* means heterozygous and *b* means homozygous haplotype 2), and genetic maps of Chromosomes 1, 2 and 12 of P1 were generated using Joinmap4.1 (Van Ooijen 2006) using the F2 population type.

## Results

### Development of an Accurate Phenotyping Protocol

In the early breeding programme of Solynta, we observed segregation for self-berry set in the first inbred populations. At that time, we assumed that this was due to segregation of the *Sli* locus; however, initial analyses revealed only a weak QTL on Chromosome 2 (data not shown). Later we realized that these populations may instead have segregated for fertility rather than compatibility.

Therefore, we improved the phenotyping protocol by accurately assessing the female and male fertility. Plants with little or no pollen were regarded as male sterile whereas plants that set neither self nor cross berries were regarded as female sterile. Sterile plants were excluded from the analysis of self-compatibility. Self-compatibility was defined as the ability of fertile plants to produce berries and seeds after self-pollination. Self-incompatibility was defined as the inability of fertile plants to set berries and seeds after self-pollination. Furthermore, to exclude pseudo self-compatibility from the analysis, we removed plants that set self-berries with fewer than 35 seeds per berry from analysis by classifying them as *Not Determined* (N.D.). While this approach allowed us to efficiently assess self-compatibility, not all plants of each population produced enough flowers to do both self and cross pollinations. For such poorly flowering plants, we visualized pollen tube growth with fluorescence microscopy of self-pollinated pistils to assign the compatibility phenotype. In our germplasm, we found that all SI plants showed pollen tube growth arrest before reaching the ovules.

### Selection of Optimal Parents for Mapping the S-locus Inhibitor Gene

Initially, the materials used for the localization of the *Sli* locus were from the diploid breeding program of Solynta. These materials were useful to select genotypes carrying the *Sli* locus and were used to generate dedicated segregating populations for our quest for the *Sli* locus. To map the *Sli* gene, we needed to identify and select genotypes with high vigour, good fertility and clear compatibility phenotypes. These were used as parents for our mapping populations.

### Selection of Self-Incompatible Parents: D2, D14 and D16 Are Fertile and Self-Incompatible

The identification of good SI parents was hampered by the fact that gradually the majority of the plants in the research and breeding programmes of Solynta were self-compatible. This prompted us to use the founders of the Solynta breeding germplasm as the best sources for SI genotypes (Lindhout et al. 2011). The founders D2, D14 and D16, showed particularly good vigour and male fertility. Clone D2 is a vigorous, highly fertile and strictly self-incompatible plant. Clone D14 is also vigorous, highly male fertile and strictly self-incompatible but has poor female fertility. Lastly, Clone D16 is vigorous and highly fertile, however, showed occasional spontaneous self-berries with some seeds; we therefore designated this clone as pseudo self-compatible.

We crossed D16 as a male to D2 and analysed the resulting F1 population to establish whether the pseudo self-compatibility of D16 is caused by a single dominant allele. We analysed the resulting population (F1(D2 × D16), *n* = 150) with the accurate phenotyping protocol. While this population consisted of 150 plants, only 51 were both male and female fertile and segregated into five SC, 21 SI and 25 plants as *Not Determined* (ND) as these did not unambiguously comply with the criteria to assess SC. In conclusion, the pseudo self-compatibility of D16 is different from the dominant self-compatibility allele of the *Sli* gene identified by Hosaka and Hanneman (Hosaka et al. 1998), it does not contain the dominant *Sli* allele and it can be used as an SI parent for mapping the *Sli* gene.

### Selection of Self-Compatible Parents: P1 and P2 Are Fertile and Self-Compatible

**Fig. 2:**
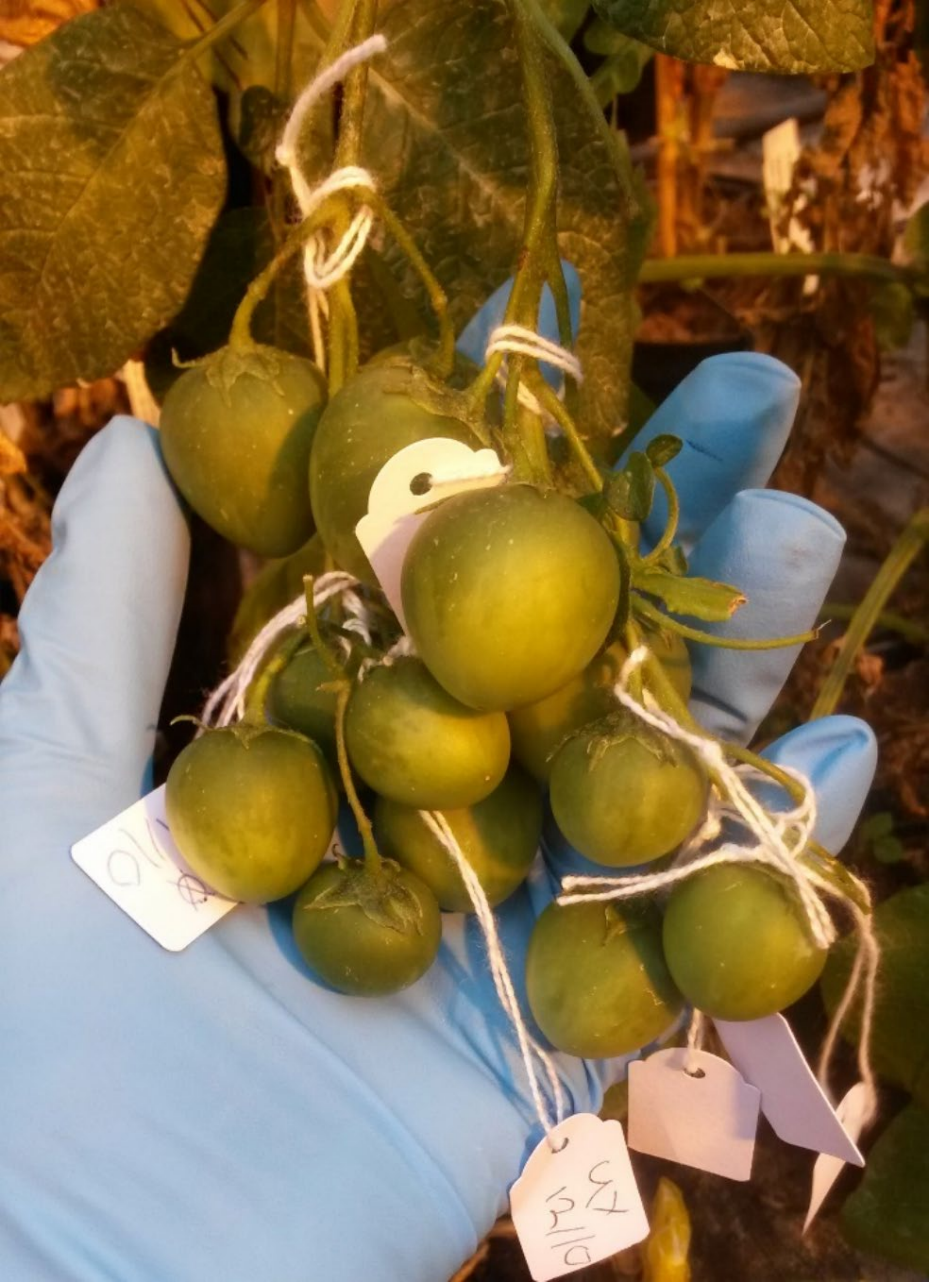
Genotype P1 readily sets many berries upon self-pollination

Next, we searched for optimal SC parents. We used the experience with the F1(D2 × D16) population and aimed to generate highly fertile populations, in which the majority of individuals could be assigned unambiguous phenotypes for SC / SI. Genotypes P1 and P2 set many berries without manual pollination. Upon vibration with an electronic toothbrush, flowers of P1 and P2 released abundant pollen and set berries containing many seeds (Fig. 2). These characters made them good candidates as SC parents. Nevertheless, we preferred to use mapping parents that were highly fertile, vigorous and more homozygous than P1. Therefore, we continued inbreeding the P1 derived progenies selecting for high self-fertility. We selected the three most self-fertile genotypes of population S1(P1), cultivated three S2 populations, S2-1(P1), S2-2(P1) and S2-3(P1) and assessed self-compatibility according to our phenotyping protocol. Interestingly, self-fertility was generally higher in the S2 populations than in the S1 population as larger percentages of plants were able to set self-berries. Furthermore, only two genotypes from population S2-1(P1) and one plant from population S2-2(P1) were classified as SI, indicating that self-compatibility was already fixed in these populations (Table 1). Finally, we selected eight of the most self-fertile genotypes from S2-1(P1) and proceeded to grow eight small S3 populations (*n* = 32 per population). Among these populations, all flowering male and female fertile plants were unambiguously SC, indicating that by selecting the most self-fertile plants in each generation, we could obtain highly fertile *Sli* homozygous plants.

**Table 1:** Fertility characteristics of F1, S1, S2 and S3 populations derived from D2, D16 and P1. The number of SI and SC plants observed in S1(P1) (denoted with an asterisk) was obtained with an earlier phenotyping protocol which did not account for male fertility. All plants in S1(P1) were genotypically SC as all had at least one copy of *Sli*

Population description				Fertility characteristics					
Population	Parents	Generation	Population size	Number of flowering plants	Number of female fertile plants	Number of male fertile plants	Number of SC plants	Number of SI plants	Number of plants of which compatibility was not determined
F1(D2 × D16)	D2 × D16	F1	150	105	76	72	5	21	25
S1(P1)	P1	S1	222	198	138	N.D	84^*^	54^*^	-
S2-1(P1)	S1(P1)-49	S2	100	100	100	100	89	2	-
S2-2(P1)	S1(P1)-88	S2	60	59	58	59	48	1	-
S2-3(P1)	S1(P1)-208	S2	60	59	55	58	38	0	-
S3-1–1(P1)	S2-1(P1)-44	S3	32	31	30	24	22	0	2
S3-1–2(P1)	S2-1(P1)-33	S3	32	25	22	24	16	0	5
S3-1–3(P1)	S2-1(P1)-82	S3	32	29	27	26	16	0	8
S3-1–4(P1)	S2-1(P1)-81	S3	32	32	32	31	27	0	4
S3-1–5(P1)	S2-1(P1)-17	S3	32	32	32	31	28	0	3
S3-1–6(P1)	S2-1(P1)-57	S3	32	30	24	28	13	0	7
S3-1–7(P1)	S2-1(P1)-10	S3	32	31	31	26	23	0	3
S3-1–8(P1)	S2-1(P1)-89	S3	32	31	29	30	25	0	3

## Selection of Optimal Populations for Mapping the S-Locus Inhibitor Gene

### Self-Fertilization of P1 Results in Extreme Segregation Distortion on Chromosome 12

In the publications identifying and mapping the *Sli* gene, Hosaka and Hanneman concluded that the *Sli* gene inherits sporophytically, meaning that the diploid genotype of the pollen donor plant determines the outcome of the pollination and implying that in a *Sli/sli* heterozygote all pollen will be able to self-fertilize and that *Sli* would segregate as a normal dominant trait (Hosaka and Hanneman 1998b, a). However, we hypothesized that the *Sli* gene acts gametophytically and that only pollen that contains the dominant *Sli* allele is able to self-fertilize. Consequently, all plants derived from the self-fertilization of a *Sli/sli* heterozygote would have at least one copy of the dominant allele and would be genotypically SC. To study the mode of inheritance of *Sli*, we generated a population from self-fertilized seeds from genotype P1 (Population S1(P1), *n* = 222, Table 1). We genotyped 186 plants with 10 KASP markers on Chromosome 1, because of the presence of the *S-locus*, and with 15 KASP markers on Chromosome 12 because of the mapping of the *Sli* gene by Hosaka and Hanneman (Hosaka and Hanneman 1998a) (Supplementary Table 3). We observed a complete absence of one homozygous genotype class on the bottom of Chromosome 12 in the same location where the *Sli* gene had previously been mapped by Hosaka and Hanneman and Peterson et al. (Hosaka and Hanneman 1998a; Peterson et al. 2016), suggesting that *Sli* inherits gametophytically and that it will not segregate in populations derived from self-fertilization (Table 2).

**Table 2:** Genotype frequencies in population S1(P1). The number of plants that are homozygous for haplotype 1 is shown under the column a, the number of plants that are heterozygous is shown under column h and the number of plants that are homozygous for haplotype 2 is shown under column b

KASP marker	a	h	b	χ2	*p* value
SOT01-22049803	45	110	27	11.4945	0.00319
SOT01-23015291	45	109	28	10.2967	0.00581
SOT01-23977865	44	112	28	11.4783	0.00322
SOT01-23999870	45	112	27	12.2174	0.00222
SOT01-30002619	45	111	26	12.7582	0.00170
SOT01-30973550	45	114	27	12.9677	0.00153
SOT01-31049683	45	113	27	12.5892	0.00185
SOT01-34981286	43	115	26	14.6413	0.00066
SOT01-35984867	45	112	26	13.1311	0.00141
SOT01-38003140	45	111	30	9.3871	0.00915
SOT12-47005400	81	86	18	43.8216	3.04972E − 10
SOT12-49992014	82	86	17	46.5892	7.64335E − 11
SOT12-50039835	82	86	17	46.5892	7.64335E − 11
SOT12-51010939	80	85	17	44.4066	2.2763E − 10
SOT12-51958661	87	82	13	61.9560	3.51905E − 14
SOT12-53002692	88	85	9	69.3736	8.62411E − 16
SOT12-53047270	88	85	9	69.3736	8.62411E − 16
SOT12-54004381	90	86	8	73.8696	9.10794E − 17
SOT12-54963278	85	95	5	69.3243	8.83933E − 16
SOT12-55015958	86	94	5	70.9784	3.86577E − 16
SOT12-55954531	87	96	3	76.0645	3.03951E − 17
SOT12-56039505	88	94	3	78.1568	1.06774E − 17
SOT12-58019020	88	97	0	84.1568	5.31598E − 19
SOT12-59975842	87	88	5	74.8000	5.71987E − 17
SOT12-61020558	83	88	13	53.6087	2.2857E − 12

### F1 Populations Derived from Reciprocal Crosses of P1 and D16 Segregate for Self-Compatibility

Based on our observations during the inbreeding of P1, we hypothesized that *Sli* acts gametophytically and cannot be mapped in inbred populations. Thus, we set out to generate segregating F1 populations by crossing P1 and P2 to SI genotypes. We hypothesized that the high fertility and vigour of the two genotypes would be inherited in the F1 populations, allowing us to score self-compatibility in a large part of the resulting progeny. We crossed P1 with D16 and grew 32 seedlings from population F1(P1 × D16) and the reciprocal population F1(D16 × P1). Both populations clearly segregated for self-compatibility, but the phenotypical segregation among those plants of which a compatibility phenotype could be assigned was significantly distorted in both populations (*χ*^2^ = 3.841 for F1(P1 × D16)^*^, *χ*^2^ = 10.6667 for F1(D16 × P1)^***^) (Table 3).

**Table 3:** Fertility Characteristics of Populations Derived from P1, D16, P2 and D14

Population description					Fertility characteristics			
Population	Female parent	Male parent	Generation	Population size	Number of flowering plants	Number of SC plants	Number of SI plants	Number of plants of which compatibility was not determined
F1(P1 × D16)	P1	D16	F1	32	32	18	8	6
F1(D16 × P1)	D16	P1	F1	32	32	20	4	8
F1(P2 × D14)	P2	D14	F1	32	32	32	0	0
BC1(P3 × D14)	F1(P2 × D14)-8 (P3)	D14	BC1	220	147	14	21	112
F1(P3 × P4)	F1(P2 × D14)-8 (P3)	F1(P1 × D16)-27 (P4)	F1	161	159	81	39	39
F1(P3 × P5)	F1(P2 × D14)-8 (P3)	F1(P1 × D16)-21 (P5)	F1	250	207	87	66	54

**Fig. 3:**
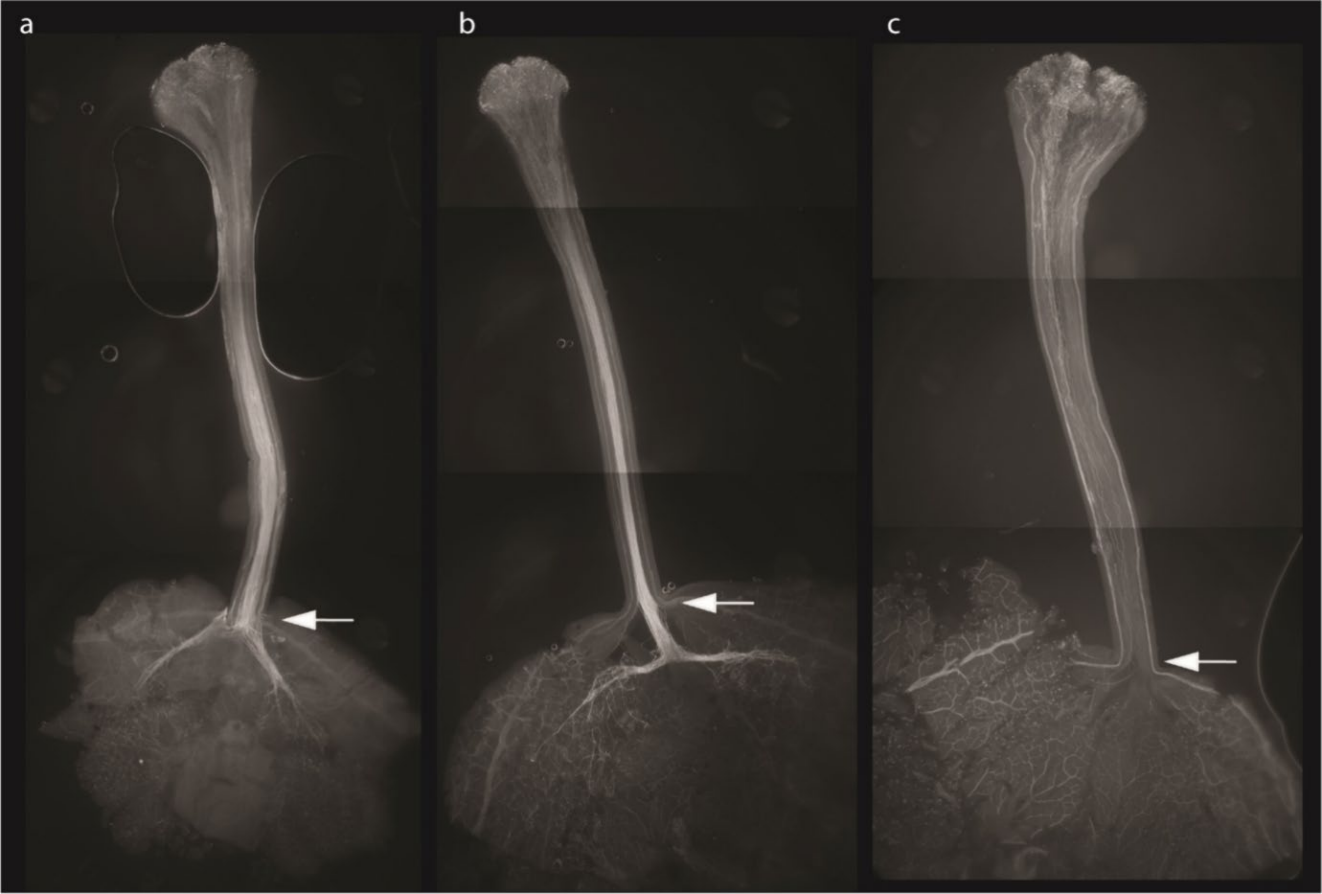
Pollen tube growth in self-pollinated pistils. White arrows point to the site where the style is attached to the ovary. Most pollen tubes of self-compatible genotypes grow through this point, whereas most pollen tubes from self-incompatible genotypes do not. **a**. Parental genotype P1 exhibits a typical self-compatible interaction, where the majority of pollen tubes are able to grow completely through the style towards the ovaries. **b**. Genotype F1(D16 × P1)-27 exhibits self-compatible interaction similar to its parent P1. **c**. Genotype F1(P1 × D16)-21 exhibits a typical incompatible interaction, the growth of the majority of pollen tubes is arrested in the style and only a few pollen tubes reach the ovaries

We visualized the pollen tube growth in the styles of all SI plants of populations F1(P1 × D16) and F1(D16 × P1), all of which showed a typical incompatible interaction, where no pollen tubes were able to grow into the ovaries (Fig. 3). By eliminating confounding sterility issues and by visualization of self-pollen tube growth of SI plants, we concluded that the two F1 populations derived from P1 and D16 segregate for the *Sli* gene, and that we could proceed with analysis of larger populations.

### F1 Populations Derived from P2, D14 and F1(P1 × D16) Segregate for Self-Compatibility

To generate an F1 population from P2, we crossed it as female to SI genotype D14. All 32 individuals of population F1(P2 × D14) were able to set self-berries with seeds, indicating that P2 is homozygous *Sli/Sli* (Table 3). We backcrossed the most fertile individual, F1(P2 × D14)-8 (afterwards referred to as P3) to D14 and assessed (self-)fertility in the resulting BC1 population BC1(P3 × D14) (*n* = 220). Unfortunately, the BC1 showed severely reduced fertility compared to the F1. Merely 173 plants flowered, and from those, the self-compatibility status of only 35 individuals could be determined based on berry set (Table 3). We decided not to proceed with further analysis of population BC1(P3 × D14), but instead chose to generate two new populations by crossing P3 to two of the most fertile SI individuals from population F1(P1 × D16), F1(P1 × D16)-27 and F1(P1 × D16)-21, referred to as P4 and P5, respectively (Fig. 1). The resulting F1 populations F1(P3 × P4) and F1(P3 × P5) were generally more fertile and segregated for self-compatibility. The genetic analysis of more individuals from population F1(P1 × D16), and of populations F1(P3 × P4) and F1(P3 × P5) allowed us to narrow down the location of the *Sli* gene on Chromosome 12, confirming the original map position of the *Sli* locus but at a smaller interval of only 169 kB (Fig. 1 and Supplementary data 1 of Eggers et al. (Eggers et al. 2021)). This has paved the way to further research on narrowing down the mapping and eventually cloning the *Sli* gene (Eggers et al. 2021).

## Discussion

### Phenotyping Protocol to Distinguish between Fertility and Compatibility

Here, we show the accurate mapping of the *Sli* gene in diploid potato, whereby accurate phenotyping methods and segregating populations are crucial. In order to map the *Sli* gene, it was critical to discriminate between general fertility effects and self-compatibility. While studying self-incompatibility in inbreeding progenies of the DM × RH population, Peterson et al. observed the same phenomenon and made a distinction between self-compatibility and self-fertility (Peterson et al. 2016). We measured male fertility by vibrating flowers with an electronic toothbrush and quantifying the released pollen. Generally, obtaining a visible amount of pollen indicates good male fertility, as in our material we did not observe plants that produced visible amounts of dead pollen due to for example tetrad sterility (Sanetomo and Nashiki 2021; Sanetomo et al. 2022). Determination of female fertility is somewhat more laborious. If a plant sets self-berries and seeds after manual pollination with collected pollen, this reliably indicates female fertility. However, for plants that are SI, cross pollinations using pollen bulked from at least three unrelated genotypes must be used to increase the chance that the bulk contains pollen with different S-alleles. One important consequence of this is that it is easier to score a plant as SC than it is to score one as SI, leading to an underestimation of the number of SI plants in a segregating population. This underestimation of SI plants can be alleviated by visualization of self-pollen tube growth using fluorescence microcopy. With this visualization, male fertility and self-compatibility can be evaluated at the same time, but it requires the removal of the styles from the flowers 48 h after self-pollination, which may affect berry set due to mechanical stress on the pistil.

### Well-Defined Parents Are a Prerequisite for Genetic Mapping

For all mapping studies in bi-parental populations, selection of suitable contrasting parents is critical. In the mapping of the *Sli* gene, parent selection is complicated by its gametophytic inheritance and the required fertility in the segregating populations. By definition, all individuals of a selfed progeny of a genotype carrying at least one copy of the *Sli* allele will inherit this *Sli* allele. Thus, selfed progenies are not useful for classical genetic mapping of the *Sli* locus, although it is possible to use the segregation distortion itself as a signal (Table 2), as was done by Ma et al. (Ma et al. 2021).

Nonetheless, our aim was to identify suitable contrasting parents to generate populations segregating for the *Sli* locus. Fertility is a crucial factor in SI parent selection, and populations whereby the majority of individual plants were highly fertile were rarely identified. Still, we identified good female fertility in population F1(P2 × D14), as all 32 individuals, including individual P3, readily set self-berries.

We observed poor fertility in BC1(P3 × D14), possibly due to inbreeding depression or specific recessive sterility factors in the genome of D14, and as such, D14 is not a perfect SI parent. In contrast, D16 is a sub-optimal parent not because of sterility but because its pseudo self-compatibility, although ultimately this did not interfere with the genetic analysis as *Sli* provides much stronger self-compatibility in terms of self-seed and berry set.

For the selection of SC parents, high fertility was also crucial. We selected two parents, P1 and P2 that showed remarkable self-berry set even without manual pollination. However, because *Sli* inherits gametophytically, it is essential that the SC parent is heterozygous for *Sli*. The only way to determine whether our SC parents were heterozygous was to make the crosses with the SI parents and observe the compatibility phenotypes in the resulting populations. Indeed, for P1, we found that its F1 populations (F1(P1 × D16) and F1(D16 × P1)) segregate for self-compatibility. In contrast, for P2, we found that all individuals of population F1(P2 × D14) were able to set self-berries, requiring that we cross one of the progenies to other SI genotypes.

### Dedicated Mapping Populations

The genotypic analysis of population S1(P1) revealed the extreme segregation distortion on Chromosome 12, supporting the hypothesis that *Sli* inherits gametophytically. However, several studies have reported the presence of lethal alleles on Chromosome 12 (Endelman et al. 2019; Zhang et al. 2019; Hosaka and Sanetomo 2020; Kaiser et al. 2021), providing an alternative explanation for the observed segregation distortion. In the founder that we used to introduce *Sli* into our breeding program (DS (Lindhout et al. 2011)), linkage between *Sli* and a lethal allele was already broken (Meijer et al. 2018). Furthermore, in another publication, we show that, in our material, segregation distortion on Chromosome 12 is indeed caused by *Sli* and that homozygosity for *Sli* in itself is not lethal (Eggers et al. 2021).

### The Quest for *Sli*: Controlling for Confounding Genetic Effects and Accounting for its Gametophytic Inheritance

*Sli* based self-compatibility is a simple monogenic trait. However, its functional expression may be obscured by several other factors, mainly related to fertility: the plants must set flowers and be male and female fertile and should preferably be reasonably vigorous. These other factors are largely determined by the genetic background of the plants, although environmental effects may have influence plant growth and fertility as well. It is the art of the geneticist to identify the effect of the desired gene among dozens of epistatic effects of other genes often each of which may only have small or moderate effects on the phenotype under investigation.

One of the main challenges of marker assisted introgression breeding is to determine the phenotypic effects of seemingly monogenic traits in multiple genetic backgrounds. Genes for which their functional expression have been clearly established in specific genetic backgrounds may not produce the same phenotypic effects when introgressed into different genetic backgrounds (Koornneef et al. 1994; Ray et al. 1995; Krüger et al. 2002; Sun et al. 2004; Hurni et al. 2014). Our quest for *Sli* was ultimately successful because we used an improved phenotyping protocol to screen parental genotypes and experimental populations for true segregation of self-compatibility instead of self-fertility by avoiding confounding fertility effects. Furthermore, the realization that *Sli* inherits gametophytically refocused our research efforts on developing segregating *F1* populations. We developed three good mapping populations derived from two SC and three SI parents. With these populations we mapped the gene to a 169 kB interval; we then used a recombinant screening approach to narrow down the interval even further and finally identified the gene by using CRISPR-Cas9 and transgenic complementation (Eggers et al. 2021). In conclusion, this study is a clear example of how the effect of a single gene can be revealed among confounding epistatic effects of other genes by the development of an accurate phenotyping protocol, the analysis of segregation distortion and the development of dedicated segregating populations. This approach has been critical in the subsequent cloning of the *Sli* gene (Eggers et al. 2021) .

## Supplementary Information

Below is the link to the electronic supplementary material.

Supplementary file1 (XLSX 38 KB)

## Data Availability

All relevant data are in the main figures and tables and in the supplementaries.
